# Meeting Report: Mode(s) of Action of Asbestos and Related Mineral Fibers

**DOI:** 10.1289/ehp.1003240

**Published:** 2011-08-01

**Authors:** Maureen R. Gwinn, Danielle DeVoney, Annie M. Jarabek, Babasaheb Sonawane, John Wheeler, David N. Weissman, Scott Masten, Claudia Thompson

**Affiliations:** 1 National Center for Environmental Assessment, Office of Research and Development, U.S. Environmental Protection Agency, Washington, DC, USA; 2 Agency for Toxic Substances and Disease Registry, Centers for Disease Control and Prevention, Atlanta, Georgia, USA; 3 Division of Respiratory Disease Studies, National Institute for Occupational Safety and Health, Morgantown, West Virginia, USA; 4 National Toxicology Program, National Institute of Environmental Health Sciences, National Institutes of Health, Department of Health and Human Services, Research Triangle Park, North Carolina, USA; 5 National Institute of Environmental Health Sciences, National Institutes of Health, Department of Health and Human Services, Research Triangle Park, North Carolina, USA

**Keywords:** asbestos, knowledge gaps, mineral fibers, mode of action, research needs

## Abstract

Background: Although asbestos in general is well known to cause a range of neoplastic and non-neoplastic human health effects, not all asbestos fiber types have the same disease-causing potential, and the mode of action (MOA) of specific types of asbestos and related fibers for various health outcomes are not well understood.

Objectives: A workshop was held to discuss the state of the science of the MOA for asbestos-related disease. The objective was to review the range of asbestos-induced health effects (including those at sites remote to the respiratory tract). We sought to identify existing knowledge gaps and define what research is needed to address these gaps and advance asbestos research.

Discussion: Discussions centered on areas of uncertainty in the field, including the ways asbestos is defined and characterized, the role of different fiber characteristics (e.g., length and mineralogy) in disease, and the impact of low-dose exposures on human health. Studying the dosimetry and mode of action of multiple fiber types would enhance our understanding of asbestos-related disease. To better elucidate the MOA of specific asbestos fibers, the risk assessor requires data as to specific characteristics of asbestos in determining fiber toxicity (e.g., surface area, mineral type), which may inform efforts to assess and control exposures and prevent adverse human health outcomes for the diverse range of fiber types. Specific research aims were defined for these topics and for overarching issues to be addressed, including the use of standardized terminology, test materials, and better experimental models to aid in data extrapolation to humans.

Conclusion: To resolve these and other issues, participants agreed that diverse scientific disciplines must coordinate to better understand the MOA leading to the various asbestos-related disease end points.

Asbestos (CASRN 1332-21-4) is a known human carcinogen [International Agency for Research on Cancer (IARC) 1977]. Some types of asbestos fibers are still in use in many parts of the world today (Azari et al. 2010; Brims 2009; Burki 2010; Park et al. 2008). Exposure to various types of asbestos can lead to fibrotic lung disease (asbestosis); a range of non-neoplastic pleural pathologies including pleural plaques, diffuse pleural thickening, pleural effusions, and rounded atelectasis; and cancer. Several cancers are considered causally related to asbestos exposure, including lung cancer, pleural and peritoneal mesothelioma, laryngeal cancer, and ovarian cancer (Sanchez et al. 2009; Straif et al. 2009). Some individual studies indicate possible associations between asbestos and other cancers, but the data are not sufficient to establish a causal link (e.g., pharyngeal, esophageal, stomach, and colorectal cancers). Numerous experimental animal studies have demonstrated the carcinogenicity of fibers of the six commercially used asbestos minerals [the serpentine chrysotile and the amphiboles cummingtonite-grunerite asbestos (amosite), tremolite asbestos, riebeckite asbestos (crocidolite), actinolite asbestos, and anthophyllite asbestos] in multiple species (rats, hamsters, mice) by exposure via inhalation, intrapleural injection, implantation, and ingestion (reviewed by Kamp 2009; Lippmann 1990). Other adverse health outcomes of asbestos exposure, including systemic effects such as autoimmune phenomena, have also been investigated recently (Blake et al. 2007, 2008; Pfau et al. 2008).

Although commercial use of asbestos has decreased in the United States, some exposures to commercial asbestos continue as old asbestos-containing building stock is renovated or demolished and as the result of continuing importation of asbestos-containing products such as brake pads and asbestos-containing cement products. In addition, as exposures to commercial asbestos have declined, exposures to noncommercial asbestos and other elongate mineral particles, often with different mineralogic characteristics and structures than fibers of the six commercially used asbestos minerals, have come into greater prominence. For example, exposures related to disturbance of contaminated soil by activities such as running, horseback riding, and use of all-terrain vehicles have been a source of concern for a number of communities (Below et al. 2011). Because there have been profound differences of opinion about whether these should be treated as asbestos exposures, they have often been highly controversial.

The general term “asbestos” was used for discussion purposes at this workshop, with clarification as to specific forms of asbestos included as needed. For ease of discussion, this general term will be used here as well to encompass asbestos and other elongate mineral fibers. Given the variability in the breadth of knowledge for specific mineral fibers, a full discussion of each type of asbestos fibers is beyond the scope of this summary report. The goal of the workshop was not to draw individual conclusions on adverse effects of specific mineral fibers, but to simply identify areas of agreement and uncertainty in the field of asbestos research in general. “Asbestos” refers to a family of elongate mineral particles with different physical and chemical characteristics. The imprecise nature of this term contributes to miscommunication and uncertainty in identifying toxicity associated with various forms of minerals, and is considered a key data gap in the field of asbestos research. Although variability exists in the potency of various mineral fibers included in this general terminology, a full discussion of these differences was beyond the scope of the workshop.

Recently, the National Institute for Occupational Safety and Health (NIOSH) released a comprehensive report on the state of the science in asbestos and other mineral fibers, with a road map for future research in the field (NIOSH 2011). The document reflected review comments on an earlier draft report prepared by a multidisciplinary panel of experts [Institute of Medicine (IOM) and National Research Council 2009]. The purpose of the document was to identify major knowledge gaps and uncertainties that needed to be resolved to allow NIOSH to update and develop evidence-based recommendations for asbestos fibers and other elongate mineral particles. This report recommends a broad research framework that can serve as a guide for development of specific research programs within and across disciplines. Recommendations included in this document also relate to the issues of terminology and definitions for asbestos and related mineral fibers (IOM and National Research Council 2009). The reader is referred to both of these documents for a comprehensive review of the issues relative to asbestos terminology and the chemical and physical characteristics of fibers involved in disease induction.

Although the NIOSH Roadmap document (NIOSH 2011) identifies toxicology research as a critical need for understanding the role of the various elongate mineral particles in disease, it does not address research elucidating the modes of action (MOAs) of asbestos and related mineral fibers in any detail. MOA encompasses a sequence of key events and processes starting with the interaction of a chemical with a cell and proceeding through various steps to disease induction. Key questions still remain in understanding asbestos-induced health effects, particularly related to the MOAs of asbestos. The MOAs of asbestos for both carcinogenic and noncarcinogenic health outcomes are not well understood. Identification of MOA for a toxic chemical or agent involves evaluation of physical, chemical, and biological information to identify key events in how an agent results in carcinogenicity (U.S. Environmental Protection Agency 2005). An environmental agent like asbestos may work through more than one MOA, with multiple MOAs for various health outcomes (e.g., lung cancer vs. mesothelioma). MOA has been used predominantly for understanding the assessment of cancer risk, but because dosimetry and other mechanisms of toxicity are shared across various toxic effects, it is applied increasingly for noncancer risk assessment as well (Bogdanffy et al. 2001; Guyton et al. 2008).

Elucidating MOAs for the various health effects induced by asbestos is critical to fully understanding the impact of mineral fiber type, dimensions, and morphology on potential to induce toxicity. Not all asbestos fiber types have the same disease-causing potential, and studying the dosimetry and mode of action of multiple fiber types would enhance our understanding of these differences. The existing known mechanistic information on deposition, phagocytosis, reactive oxygen species (ROS) production, and gene expression alterations induced by asbestos exposure has been observed in both *in vitro* and *in vivo* research studies, but in many cases the role of these and other biological alterations as key events in human pathology and diseases are still being elucidated. A particular knowledge gap for understanding modes of action for nonpulmonary health end points is clarification of the relative roles of direct effects of fiber translocation to extrapulmonary targets versus indirect systemic effects of fiber exposure mediated by agents such as cytokines or growth factors.

The NIEHS, along with other federal agencies, held a workshop to address the current state of the science as it relates to the MOAs of asbestos and other related mineral fibers. Six main topic areas were defined before the workshop: health outcomes (pulmonary, pleural, and nonpulmonary), mutagenicity, susceptibility, and low-dose exposure response to fibers (Appendix 1). Based on their publication record and knowledge of the field, expert researchers were contacted by the workshop planning committee and asked to select and lead teams of experts to provide written reviews for each topic area for discussion at the workshop. Workshop discussants included these writing teams, invited reviewers recommended by these teams, and public observers. The complete reviews of each of these topics are published separately and include knowledge gaps and specific research needs (Aust et al. 2011; Below et al. 2011; Broaddus et al. 2011; Bunderson-Schelvan et al. 2011; Case et al. 2011; Gwinn 2011; Huang et al. 2011; Mossman et al. 2011). Several cross-cutting issues were defined across the topic areas. Of particular interest were delineating areas of broad consensus versus areas of uncertainty. These issues and highlights of the workshop are summarized below.

## Workshop Summary

Pulmonary disease after exposure to asbestos is a well-studied area of asbestos-induced diseases. The MOAs of asbestos-induced pulmonary effects appear to be different for the various adverse health outcomes (lung cancer, asbestosis, mesothelioma) and the various fiber types. An understanding of MOAs is hindered by potential confounding factors in human studies and limited characterization of fibers in most laboratory animal studies (Mossman et al. 2011). These limitations in fiber characterization contribute to some of the debate regarding the role of various fiber types and sizes in disease and make mechanistic comparisons between fiber types difficult. One example is the continued controversy on the role of short fibers of various asbestos types in producing disease (Aust et al. 2011; Case et al. 2011; Mossman et al. 2011).

Along with mesothelioma, asbestos exposure is associated with non-neoplastic interstitial disease and pleural pathologies (e.g., plaques, diffuse pleural thickening, and effusions). Despite the known association between asbestos exposure and pleural responses, questions still remain on the fundamental process of translocation of fibers to the pleural cavity. One experimental issue concerns the dissimilarities between the pleural cavities of different species. Despite these differences, much has been learned about the mechanisms of pleural disease, particularly the interactions between fibers and target cells, both in the pleural cavities of laboratory animals and in *in vitro* studies using both animal and human cells (Broaddus et al. 2011).

Nonpulmonary effects after asbestos exposure are not well understood. Strong support exists for an association between asbestos exposure and intra-abdominal processes such as peritoneal mesothelioma and ovarian carcinoma (Straif et al. 2009). There is also some support, although data are limited, for autoimmune effects associated with asbestos exposure (Bunderson-Schelvan et al. 2011). The IARC concluded recently that the evidence linking asbestos exposure to stomach cancers is limited (Straif et al. 2009). Asbestos exposure may potentially lead to other nonpulmonary diseases; however, very few data exist to establish a causal relationship (Bunderson-Schelvan et al. 2011).

One of the more debated issues related to asbestos is whether specific asbestos fibers are mutagenic. Mutagenicity is a specific term that refers to a permanent, heritable change in the structure or amount of genetic material of an organism and includes gene mutations as well as structural and numerical alterations in chromosomes (Eastmond et al. 2009). Genotoxicity is a broader term, referring to the ability of a substance to damage DNA and/or cellular components that regulate the fidelity of the genome (e.g., spindle apparatus, DNA repair systems) (Eastmond et al. 2009). Although there is general agreement that some types of asbestos are genotoxic *in vitro*, either directly (i.e., fiber interactions with the spindle apparatus) or indirectly (i.e., ROS production), there is less agreement on the mutagenicity of asbestos fibers, particularly *in vivo*. Most genotoxicity studies of various types of asbestos have been performed *in vitro*, and therefore limited *in vivo* data are available to address this issue. An in-depth review of the existing literature (Huang et al. 2011) suggests a role for mutagenesis in asbestos-induced neoplastic diseases but not in non-neoplastic diseases. Thus, mutagenicity is one MOA for asbestos-induced neoplastic diseases, although this does not rule out a role for inflammation, cellular toxicity, and oxidative stress. These latter modes of action are believed to be involved in non-neoplastic disease as well.

Another area of increasing investigation is susceptibility to asbestos-induced diseases, particularly malignant mesothelioma (MM). There are limited studies examining the impact of age, sex, ethnicity or genetic makeup, disease status, and nutrition on asbestos-induced diseases. MM generally occurs late in life, and predominantly in men. However, because the majority of epidemiology studies are on male-dominated occupational populations, generally fewer females have been studied. The long latency period of MM is suggestive of multiple somatic genetic conversions after exposure to asbestos. Ionizing radiation (Goodman et al. 2009) and some viral exposures (Yang et al. 2008) have also been associated with susceptibility to MM. Recent studies have also highlighted key biomarkers for MM, including soluble mesothelin-related peptide/megakaryocyte potentiating factor (both encoded by the same gene) (Cristaudo et al. 2007; Pass et al. 2008; Scherpereel and Lee 2007), osteopontin (Scherpereel and Lee 2007), and MN/CA9 (Li et al. 2007). Unfortunately, no one marker is specific to this tumor type. Ugolini et al. (2008) recently reviewed 20 reports of familial MM and determined that there is likely a polygenic component to this disease.

## Discussion

The workshop participants highlighted some overarching issues that were considered obstacles to progress in asbestos research. These include inconsistency in the use of the definition of asbestos fibers, a lack of standard size-selected reference materials, and an inadequate understanding of appropriate dose metric(s). Further, there is a need to better understand *a*) the potential for fiber-induced mutations that influence cancer, *b*) relevant mechanisms of translocation of different fiber types to extrapulmonary sites, *c*) health effects outside of the respiratory tract and pleural tissue, and *d*) the role of population variability and sensitivity to asbestos-induced adverse health end points. The variability in approach to each of these areas impairs the ability to compare results across studies. Each overarching issue discussed is briefly described below and includes broad recommendations for future research in fiber toxicity.

***Standardized terminology.*** The need for standardized terminology is paramount. There is no consensus on how to define terms as basic as “asbestos” and “fiber.” Agreement between the many disciplines and researchers interested in understanding the environmental (human and ecologic) effects of asbestos exposure would greatly facilitate progress, because inconsistent use of terminology currently makes it difficult to compare results across the many existing studies. Recommendations from this workshop include that until standardized definitions are in place, researchers should at a minimum be encouraged to explicitly state their definitions and provide full fiber size distributions for the materials used in experimental studies. The recent NIOSH Roadmap report (NIOSH 2011), in response to the NRC review (IOM/National Research Council 2009) of the draft document, discusses issues related to appropriate terminology to be used in describing various types of asbestos fibers.

***Sample characterization/measurement methods.*** Another overarching issue is the need for more explicit reporting of findings with respect to both definition of fiber and the type of sampling methods, including how fibers were quantified. Counting rules need to be well defined for results to be compared consistently and correctly between analytical laboratories. Counting rules include the fiber dimensions selected as the cutoff for inclusion in the fiber count, the total number of objects to be counted, and the methodology used. This is necessary to be able to rigorously compare results across studies, and relates to limits of detection and how these limits may lead to truncation of fiber distributions that may be implicit in different experimental designs. There was a strong general agreement that it is necessary to perform a complete characterization of all samples being compared and to fully describe this information, including counting rules, when publishing the results of a study. There are still some questions as to what constitutes a full characterization and which methods are best suited to use for these purposes.

***Standard size-selected reference materials.*** It was recognized that the use of standard reference materials is also needed to assist in comparing research from multiple laboratories and test conditions. However, even if standard reference materials were made available for research, standard definitions for consistent characterization of materials used in research studies are still needed, as mentioned above. Size-selected test materials also are needed to disaggregate the influence of fiber dimension, morphology, and mineral form on specific toxic actions of fibers observed in both *in vitro* and *in vivo* experimental systems.

***Defining the appropriate dose metric.*** Another key issue discussed by the workshop participants was related to identifying the appropriate dose metric to be used in asbestos studies. As discussed above, there are key knowledge gaps regarding the role of physicochemical characteristics in asbestos-induced health effects. This makes the choice of a dose metric for any response analysis extremely important. Even though it is not clear which characteristics are important for understanding relative differences between fiber types, it is clear that the standard use of mass alone is probably not sufficient. Comparisons between fiber types based solely on an equal mass basis do not take into account differences in fiber number, surface area, reactivity, or fiber dimensions in each sample. This can lead to erroneous conclusions about the relative potencies of fiber types, which can have serious ramifications. Rather than prescribe the definition of a dose metric *a priori*, studies should explore various dose metrics to ascertain which one best describes the exposure–response relationship for internal burdens at the organ or cellular levels as well as the exposure–response relationship for the given end point or outcome measure under evaluation.

***Development of novel experimental systems.*** One of the primary reasons that uncertainties exist in understanding the mechanistic response to asbestos is the lack of appropriate *in vivo* and *in vitro* test systems. There is a continued need for experimental systems that better reflect the MOAs for asbestos-induced disease in humans, specifically for pleural effects and translocation of fibers to the pleural space, as well as tissue-specific mutagenicity assays. More research is needed with appropriate target cells (i.e., mesothelioma cells, Clara cells, alveolar epithelial cells) that is focused on the biological role those cells may have on the pathology of disease.

***Genotoxicity of asbestos.*** The role of genotoxicity in asbestos-related diseases is unknown. Further, although existing data suggest that alterations in gene expression and epigenetic effects may contribute to some types of asbestos fibers inducing disease, more research is needed to fully understand the complex and overlapping signaling pathways involved in these effects, including ROS production, DNA damage and repair and p53 activation, cell death (apoptosis or necrosis), and inflammation. The interaction of these and other alterations leading to fiber-induced mutagenicity is unclear. Another key issue related to cancer risk assessment of asbestos is whether there is a possible threshold exposure level (duration and magnitude) below which there is no genotoxic response after fiber exposure. Therefore, well-conducted *in vitro* and *in vivo* studies are needed to clarify this controversial issue.

***Extrapolation to humans.*** It was recognized that extrapolation of results from *in vitro* studies and *in vivo* laboratory animal studies to human populations is an important knowledge gap and research need. Although well-designed epidemiologic studies examining malignant and nonmalignant health effects of a range of fiber types after multiple routes of contemporary exposure would be ideal for understanding pathogenicity in humans, such populations are fortunately now rare. Furthermore, if identified, it would be unethical even to allow such exposure conditions to persist. In addition, human epidemiology studies are generally hindered by limited exposure information. Therefore, validated laboratory animal models (including primates) will be necessary to better understand the dose response for various asbestos-related disease outcomes. These laboratory animal models must be demonstrated to be relevant to human asbestos-induced adverse health effects to improve our understanding of pathogenesis and develop useful biomarkers.

***Population variability and sensitivity.*** There is a major gap in understanding individual susceptibility to the various types of asbestos-induced disease and variability in susceptibility across populations. Limited data are available regarding the impact of asbestos exposure in children, and the role of many preexisting health conditions on asbestos-induced disease is unknown. Although the impact of certain coexposures such as smoking has been known for decades (Selikoff et al. 1968), limited information is available on the role of other coexposures.

## Conclusions

Even after decades of research, and numerous publications worldwide, the workshop participants concluded that there are still major knowledge gaps hindering better understanding of the MOAs of various types of asbestos fibers.

Workshop participants agreed on a key overarching question: Is there sufficient understanding of the mechanisms of asbestos toxicity to determine the relevance of mineralogy and dimension in asbestos-induced disease? More specifically, do fibers < 5 µm in length have biological activity, and is there differential toxicity between serpentine and amphibole fibers? Although it is clear that fiber physicochemical characteristics play a role in asbestos-induced disease, it is less clear which characteristics are important to specific adverse health effects. In particular, discussion focused on the role of short fibers in disease. There are many researchers in the field who believe there is sufficient scientific evidence to show that short fibers (< 5 µm) do not play a role in inducing disease. However, others disagree with this conclusion. Similarly, consensus was not reached on the relative potency of different fiber types. Current knowledge regarding the characteristics that contribute to differences in relative potency between fiber types is incomplete, with a key research need being to systematically evaluate different fiber types. Participants agreed that although there is a wealth of information on mechanisms of asbestos-induced lung cancers, there is limited mechanistic understanding of how fiber mineralogy, dimensions, surface reactivity, and biopersistence contribute to all asbestos-induced diseases, particularly nonpulmonary end points. Additional studies, both *in vitro* and *in vivo*, are needed to determine the role of specific physicochemical characteristics from multiple fiber types in adverse health effects after exposure to asbestos and related mineral fibers.

One of the main objectives of this workshop was to bring together experts from diverse fields to encourage cross-fertilization of ideas to advance the field of asbestos research. Breakout group discussions from this workshop resulted in future multidisciplinary research ideas to address many of the existing knowledge gaps in the field. These included a follow-up workshop on standardized terminology, increased inclusion of geologists in the planning and implementation of toxicology and epidemiology studies, and the need for increased research funding for large-scale integrative multidisciplinary laboratory studies.

In conclusion, despite the breadth of the available literature on asbestos-induced health effects, unanswered questions still remain. The lack of clear answers may be attributable partly to different interpretation of the existing data, which can be related to many of the overarching research needs described above (rigorous attention to use of standardized terminology, sampling characterization/measurement methods, use of standard reference materials, and use of appropriate dose metrics). This workshop and the resulting state-of-the-science reviews seek to advance our understanding of the health hazards of different types of asbestos fibers by identifying *a*) key knowledge gaps and *b*) the research needed to address these gaps to more fully understand determinants of toxic responses to asbestos and how asbestos exposures cause disease.

## Appendix 1. Key topic areas for discussion at Asbestos MOA Workshop.

Based on a mechanism/MOA, can we determine the range of fibers or specific components that contribute to adverse health effects, keeping in mind the role of different fibers in various disease end points. A key consideration will be how to express the dose metric corresponding to different effect end points, e.g., fiber burden (milligrams or surface area) normalized to lung tissue volume.
Pulmonary end points (cancer and noncancer)
  Deposition/translocation to the target organ.
    Mechanisms required for fiber internalization by the cell (by fiber size).
    Disease end points (mechanisms/MOA, fiber determinants of toxicity).
    Any fibers considered inactive for a particular end point and why.
    Dose metric options and issues of adjustment for sensitivity.
  Pleural end points (cancer and noncancer)
  Deposition/translocation to the target organ.
    Mechanisms required for fiber internalization by the cell (by fiber size).
    Disease end points (mechanisms/MOA, fiber determinants of toxicity).
    Any fibers considered inactive for a particular end point and why.
    Dose metric options and issues of adjustment for sensitivity.
  Nonpulmonary end points (cancer and noncancer)
  Deposition/translocation to the target organ.
    Mechanisms required for fiber internalization by the cell (by fiber size).
    Disease end points (mechanisms/MOA, fiber determinants of toxicity).
    Any fibers considered inactive for a particular end point and why.
    Dose metric options and issues of adjustment for sensitivity.
    The role of fibrosis in carcinogenicity.
Genetic toxicology
Role of mutagenicity in fiber-induced carcinogenicity
  Is there a mutagenic MOA?
    Mutagenicity/carcinogenicity at low doses.
    Appropriate dosimetric for biological activity leading to mutagenicity and impact of target cell/tissue type.
    Health end points with and without mutagenicity as a key event.
    Influence of determinants of toxicity in mutagenicity.
Susceptibility
Main factors that impact susceptibility to fiber-induced health effects
  Age
    Genetics
    Disease status
    Nutrition
    Sex
    Lung architecture/ventilation patterns.
Extrapolation of environmental exposure levels (low dose, sporadic, high dose) to health effects.
Role of lung exposure levels/regimen/determinants of toxicity
  Lung architecture/ventilation patterns
    Deposition, clearance, and overload
    Inflammation
    Mechanism of toxicity below inflammatory response.
